# Current and Emerging Therapies in the Management of Hypoxic Ischemic Encephalopathy in Neonates

**DOI:** 10.3390/children5070099

**Published:** 2018-07-19

**Authors:** Jayasree Nair, Vasantha H.S. Kumar

**Affiliations:** Division of Neonatology, Jacobs School of Medicine and Biomedical Sciences, University at Buffalo, Buffalo, NY 14203, USA; jnair2@buffalo.edu

**Keywords:** birth asphyxia, hypoxic ischemic encephalopathy, neonatal encephalopathy

## Abstract

Neonatal hypoxic ischemic encephalopathy (HIE) presents a significant clinical burden with its high mortality and morbidity rates globally. Therapeutic hypothermia (TH) is now standard of care for infants with moderate to severe HIE, but has not definitively changed outcomes in severe HIE. In this review, we discuss newer promising markers that may help the clinician identify severity of HIE. Therapies that are beneficial and agents that hold promise for neuroprotection are described, both for use either alone or as adjuncts to TH. These include endogenous pathway modifiers such as erythropoietin and analogues, melatonin, and remote ischemic post conditioning. Stem cells have therapeutic potential in this condition, as in many other neonatal conditions. Of the agents listed, only erythropoietin and analogues are currently being evaluated in large randomized controlled trials (RCTs). Exogenous therapies such as argon and xenon, allopurinol, monosialogangliosides, and magnesium sulfate continue to be investigated. The recognition of tertiary mechanisms of brain damage has opened up new research into therapies not only to attenuate brain damage but also to promote cell repair and regeneration in a developmentally disorganized brain long after the perinatal insult. These alternative modalities may be especially important in mild HIE and in areas of the world where there is limited access to expensive hypothermia equipment and services.

## 1. Introduction

Significant progress has been made globally in reducing mortality in children under five years of age following the United Nations Millennium Declaration, signed in 2000. Millennium developmental goals (MDG) 4 and 5 specifically focus on reducing child mortality and improving maternal health [1]. Despite the decline in under five mortality by 49% to 46/1000 live births, there is a much slower decline in neonatal mortality, which constitutes about 45% of all childhood deaths. The devised action plan of sustainable development goals aims to reduce neonatal mortality to 12/1000 and under five mortality to 25/1000 live births by 2030 (https://sustainabledevelopment.un.org/sdg3).

The most common contributor to early neonatal mortality is birth asphyxia with prematurity, infections, and low birth weight being other major contributors. Four million newborn infants experience birth asphyxia each year, accounting for an estimated one million deaths and 42 million disability-adjusted life years [2]. Many of these infants sustain significant brain injury and develop long-term sequelae, most commonly cerebral palsy, epilepsy, and sensory deficits [2]. Advances in managing infants with birth asphyxia, leading to hypoxic ischemic encephalopathy (HIE) on a global scale will contribute significantly to achieving the 2030 sustainable developmental goals.

With the advent of therapeutic hypothermia (TH), improved outcomes are being reported in moderate HIE. TH, however, has not demonstrated improvement in outcomes related to severe HIE. As hypothermia, both whole body and head cooling, is being evaluated and used across the globe, several limitations for its use, related to accessibility, provision of adequate facilities for initiation and monitoring hypothermia and financial limitations—especially in developing countries—are being recognized. This has led clinicians and researchers to continue evaluating complementary and/or alternative therapies for infants with HIE. In this review, we will discuss current and emerging therapies in the management of HIE, other than hypothermia.

## 2. Current Standard of Care

Any pregnancy that is identified as being at high risk for neonatal complications should ideally be delivered at a tertiary care center with trained and experienced resuscitators. Management of an infant who is depressed at birth involves following accepted guidelines such as those published by ILCOR and Neonatal Resuscitation Program [3]. The infant is evaluated for hypothermia, which should ideally commence within 6 h of birth for infants with moderate to severe HIE [4]. Improved motor outcomes have been noted with earlier cooling within 3 h after birth [5]. Supportive management of seizures, fluid balance, and hematological and cardiovascular abnormities is essential in ensuring optimal outcomes [6]. Presence of a multidisciplinary team including pediatric neurologists, cardiologists, and other subspecialties as well as institutional capabilities for long term EEG, MRI, and physical and occupational therapies are a requisite for establishment of a cooling protocol at tertiary institutes [6]. Follow up with a developmental pediatrician and enrollment in Early interventional programs are also essential to optimize outcomes for infants with HIE [7].

## 3. Pathophysiology of HIE and Implications for Intervention

The primary pathophysiologic event that results in HIE is impaired oxygen delivery to the brain tissue. This could be caused by reduced oxygen carrying capacity primarily from the hypoxic component or from reduced cerebral blood flow due to volume loss or impaired circulation. Often, a combination of these events is seen. While in some infants the exact causative event cannot be identified, the pathophysiology of brain injury in HIE is well described in term and near term infants.

**Figure 1:**
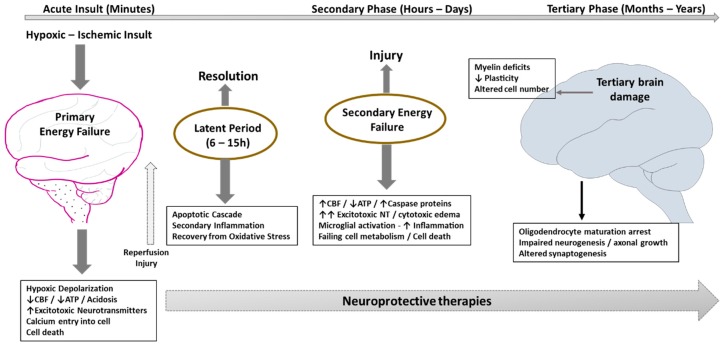
Schematic illustration of pathophysiology of HIE in relation to hypoxic ischemic (HI) insult resulting in primary (acute phase) and secondary energy failure (secondary phase) in the brain. Brain damage (tertiary phase) continues to occur months to years after the injury resulting in decreased plasticity and reduced number of neurons. Latent period following resuscitation is ideal for interventions to decrease the impact of secondary energy failure. However, strategies are developed to attenuate tertiary brain damage which will expand the therapeutic window, substantially increasing the beneficial effects of neuroprotection in these infants and hence its impact on long-term outcomes. CBF—cerebral blood flow; ATP—Adenosine tri phosphate; NT—neurotransmitters.

Primary energy failure occurring from the initial hypoxic ischemic (HI) insult (Figure 1) is characterized by decreased ATP production and systemic acidosis from increased lactate. Energy failure at the cellular level leads to loss of integrity of the neuronal cell membrane, with calcium entry into the cell facilitated by activation of NMDA receptor and other excitotoxic neurotransmitters. There are very few intervention strategies at this stage to limit damage from the primary energy failure that occurs within minutes to hours of the initial insult. However, a period of latency lasting several hours follows primary energy failure, which opens up the window of opportunity for interventions to limit further neuronal damage. In the absence of any intervention, secondary energy failure ensues in moderate to severe HIE. However, the degree of resuscitation following HI injury and the severity of primary energy failure mechanisms may impact not only the latency period, but also the subsequent changes in secondary energy failure in the brain. Secondary energy failure typically occurs 6 to 48 h following HI insult and appears to be related to oxidative stress, inflammation, excitotoxicity, and ultimately to cell death. In a subset of infants, persistent active mechanisms may prevent regeneration of neurons or exacerbate brain damage resulting in tertiary brain injury [8]. Tertiary damage to the neurons—such as myelin deficits, reduced plasticity, and altered cell number—could persist for months to years after the initial insult. Potential mechanisms that oversee these effects include persistent inflammation and epigenetic changes resulting from blockade of oligodendrocyte maturation, impaired neurogenesis, impaired axonal growth, or altered synaptogenesis [9,10].

Timely clinical assessment is essential to facilitate intervention in the latent period. Anticipation from prenatal history and risk factors prepares the team for potential HIE. A detailed neurological exam helps the clinician determining the severity, generally classified as mild, moderate or severe HIE. TH is typically started within 6 h of birth for it to be effective, before secondary energy failure ensues. However, the concept of tertiary brain injury and alternate therapies that could continue to affect prognosis has expanded the treatment window of HIE. Despite advances in management options, it is difficult to predict HIE in infants, as some of them develop HIE in spite of a normal neurological exam. Recently, specific biomarkers of neuronal injury are being explored for earlier identification of HIE in these infants.

## 4. Evaluation of HIE: Emerging Modalities

### 4.1. Biomarkers

Due to the varied clinical presentation of HIE, it is difficult to predict outcomes early during the course, when intervention with targeted therapies would have maximal effect. Hence, biomarkers that could help detect presence and severity of HIE are being evaluated. These are chemicals that are measurable in blood, urine, cerebrospinal fluid (CSF), or from multiple locations reflective of brain injury or regeneration. Currently, there are no easily available and specific biomarkers for clinical use. In this section, we will describe emerging biomarkers in HIE.

#### 4.1.1. Specific Biomarkers of Brain Injury

S100B is a calcium binding protein released by brain glial cells in response to injury [11]. Elevated levels of S100B have been evaluated in cord blood [12], urine [13], CSF, as well as amniotic fluid for newborns with HIE or encephalopathy. A recent clinical study elevated umbilical cord blood levels of this protein in neonates suffering from HIE stages II-III, suggesting that this biomarker may correlate with the severity of disease and the risk of adverse neurodevelopmental outcomes and/or death [12]. Elevated S100B protein in plasma within 24 h after birth is associated with increased brain injury as evaluated by MRI in a cohort of 50 newborns with HIE [14].

Neuron specific enolase (NSE) is a glycolytic isoenzyme that is released after neuronal death. Celtik et al. identified NSE cut off values that could help differentiate mild moderate and severe encephalopathy [15]. Another recent study correlated NSE levels in the early postnatal period with poor outcomes at two years of age [16]. However, other studies evaluating plasma biomarkers in HIE have found no correlation between NSE levels and brain injury outcomes [14,17]. Additionally, NSE is affected by any hemolytic process which is a drawback to its utility as an effective biomarker [18].

Glial fibrillary acidic protein (GFAP) is released with damage to astroglial cells and levels in blood and CSF have been seen to correlate with severity of HIE, MRI changes, and developmental outcomes in small studies [19,20]. However, there are no norms for GFAP values in various body fluids and a recent study in newborns with HIE found very low GFAP levels, below the assay lower limits for detection [14].

Several other biomarkers of brain injury including ubiquitin carboxyl-terminal hydrolase L1 (UCH-L1) and total tau protein also hold promise as early biomarkers of brain injury in HIE that are being studied further [14,21].

#### 4.1.2. Inflammatory Markers

Several cytokines are released in acute HIE and their potential as early and accurate biomarkers is being extensively researched. Interleukin (IL)-6 and IL-16 in cord blood were found to be significantly associated with electrographic and clinical HIE severity in a study of full term infants [22]. In another study, plasma levels of IL-6, IL-8, and vascular endothelial growth factor was associated not only with severity of HIE but also predicted abnormal neurological outcomes [20]. However, inflammatory cytokines continue to be limited in their lack of specificity, with other causes of inflammation such as infections and hemolysis also producing a cytokine response.

### 4.2. Metabolomic Analysis and Metabolites

Metabolomic analyses are being used in various conditions to identify its presence in time for appropriate therapy. Distinct patterns of changes in body metabolites have been evaluated in a recent metabolomic analysis in a non-human primate model of HIE. Eight metabolites (arachidonic acid, butanoic acid, citric acid, fumaric acid, lactate, malate, propanoic acid, and succinic acid) were noted to correlate with adverse neurodevelopmental outcomes [23] in this model. While promising, larger studies evaluating metabolomics are required before this can be used for clinical prognostication.

### 4.3. Imaging

Magnetic resonance spectroscopy (MRS) has significantly improved our understanding of the changes in brain metabolism and evolution of brain injury after a hypoxic ischemic insult. It could help define the severity of injury if done during the treatment window (within 6 h) by estimating phosphocreatine (PCr) and cerebral lactate or inorganic phosphate. High PCr has been associated with a favorable outcome [24] while high lactate and Pi may indicate a poor outcome [25]. MRS after 6 h continues to provide useful clues to help the clinician prognosticate HIE insult. Low cerebral PCr/Pi, high cerebral lactate, and decreased nucleoside triphosphate are associated with poor outcomes. Persisting brain lactic acidosis on MRS is seen in infants with poor neurodevelopmental outcomes through the first year after birth [26].

## 5. Emerging Therapies

**Figure 2:**
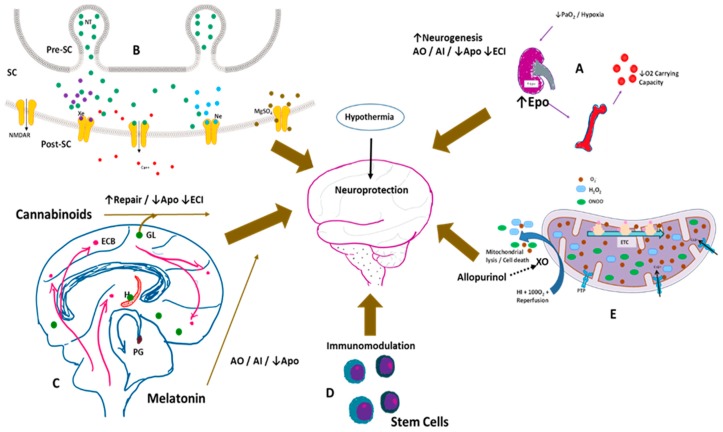
Potential neuroprotective therapies in the management of hypoxic-ischemic encephalopathy. Hypothermia is currently the standard of care in the management of moderate to severe HIE in infants. (**A**) Hypoxia stimulates erythropoietin (Epo) production by the kidneys, increasing RBC production in the bone marrow, thereby increasing O_2_ carryign capacity. Epo favors neurogenesis; is an antioxidant (AO), anti-inflammatory (AI), decreases apoptosis (Apo) and excitotoxic cell injury (ECI); (**B**) Xenon (Xe—purple dots), neon (Ne—teal dots), and magnesium sulphate (Mgso4—brown dots) antagonize the N-methyl-D-aspartate (NMDA) mediated excitotoxicity via the NMDA receptor (NMDAR), decreasing calcium entry into the cell. SC—synaptic cleft, Pre-SC—presynaptic cleft, Post-SC—postsynaptic cleft; (**C**) Endogenous cannabinoids (ECBs) and melatonin are neuroprotective. Spingolipids such as gangliosides (GL) also protect against apoptotic injury; (**D**) Umbilical cord blood derived stem cells and mesenchymal stem cells modulate the immune system and affect long-term outcomes; (**E**) Allopurinol by inhibiting the enzyme xanthine oxidase (XO), decreases reactive oxygen species, mitochondrial lysis, and cell death. O_2_—superoxide, H_2_O_2_—hydrogen peroxide, ONOO—peroxynitrite, ETC—electron transfer chain; PTP—permeability transition pore of inner mitochondrial membrane.

Most of the neuroprotective strategies being evaluated for use in management of HIE (Table 1, Figure 2), are primarily to mitigate the devastating effects from secondary energy failure on the brain. Therapies that are applied in experimental and animal models of HIE generally work on slowing the pathophysiology that include decreasing oxidative stress, antagonizing excitatory neurotransmitter release or receptor blockade, anti-inflammatory effects, immunomodulation or by decreasing apoptosis among others. While some are undergoing randomized controlled trials such as erythropoietin and its analogues, others are being researched in experimental animal models. Several others have been evaluated anecdotally.

### 5.1. Resuscitation

#### 5.1.1. Optimizing Placental Transfusion

Either by delayed cord clamping or by the process or cord milking/stripping may impart a neuroprotective effect, besides improving hemodynamics [27]. Full-term infants who underwent DCC had a 45% higher mean ferritin concentration at four months of age [28], demonstrating a beneficial effect in preventing iron deficiency anemia. Iron plays an important role in brain myelination [29], suggesting a neuroprotective effect of placental transfusion. Additionally, umbilical cord derived stem cells are being evaluated for treatment of HIE, and ensuring an optimal placental transfusion ensures that the compromised infant receives the entire complement of stem cells.

#### 5.1.2. Vasopressin

Use of vasopressin as an alternative to epinephrine in neonatal resuscitation is being evaluated in pre-clinical studies. This medication has a neuroprotective potential as studies in rat and guinea pig have demonstrated that vasopressin activates hippocampal interneurons, silencing synchronous neuronal activity [30]. This may reduce neuronal energy demand, which could be a neuroprotective mechanism.

### 5.2. Erythropietin/Analogues (Endogenous)

Erythopoietin (Epo) is an endogenous protein, synthesized in the fetal liver that has an impact on multiple critical pathways. Besides stimulating erythropoiesis, Epo is a cytokine that influences the body’s immune response [31]. Additionally, its neuroprotective role has been recognized and evaluated in pre-clinical and clinical studies. Epo receptors (EpoR) are widely expressed throughout the central nervous system in several cell types including progenitor cells, astrocytes, oligodendrocytes, and microglia [32,33], to name a few. Epo and EpoR are upregulated following hypoxic ischemic injury and Epo has an anti-oxidant [34] as well as anti-inflammatory [35] effect. It reduced apoptotic [36,37] and excitotoxic cell injury [38]. Clinical trials evaluating Epo alone [39,40] in infants with HIE have shown promising results. Since hypothermia has become standard of care therapy for HIE, larger trials are currently on going evaluating Epo as a complement to cooling therapy. A Phase I trial evaluating effective dose and safety [41] demonstrated that a moderately high dose of 1000 U/kg achieved levels (based on animal studies) that would protective maximal neuroprotection and minimize risks of excessive Epo. In a Phase II double-blinded, placebo-controlled trial in infants undergoing TH for HIE, multiple doses of Epo (1000 U/kg) resulted in less MRI brain injury and potential for improved short-term motor outcomes [42]. A Phase III trial evaluating the effect of Epo with TH on the combined outcome of death or neurodevelopmental disability is currently underway [43].

Darbepoietin (darbe) is a long acting erythropoietin analogue that offers the additional benefit of once weekly administration. A small randomized trial evaluating the use of darbe as adjunctive therapy to hypothermia in the first 12 h of life and repeated at one week of life demonstrated a good safety profile of this medication [44]. A large multicenter randomized controlled trial evaluating use of darbe in mild HIE is currently underway (MEND study ClinicalTrials.gov Identifier: NCT03071861).

### 5.3. Stem Cells

Recent experimental studies in animal models have indicated that various mechanisms of action are involved in the process by which umbilical cord blood cells (UCBCs) protect the brain from hypoxic ischemic injury. These stem cells are predominantly derived from two sources—bone marrow derived mesenchymal stem cells (BM-MSC) and umbilical cord blood derived mesenchymal stem cells (UCB-MSC) [45]. Preclinical studies have been done evaluating stem cells in neonatal brain injury. Cord blood stem cell therapy has could have protective effects mainly on inflammation, apoptosis, oxidative stress, and may enhance regeneration [46]. Unlike hypothermia, cell based therapy may provide a longer therapeutic window as repair and regeneration take place over longer periods of time. Donor MSCs do not last for a long time in the brain, but do have paracrine effects by secreting trophic and immunomodulatory factors that help in repair from brain injury. A meta-analysis of MSCs demonstrated a significant positive effect on neurobehavioral outcome following HIE injury [47]. Specifically in a preterm model of global hypoxic injury, IV mesenchymal stem cells reduced cerebral inflammation by induction of T-cell tolerance [48]. A Phase I clinical trial evaluating UCB cells to neonates with HIE showed that collection, preparation, and infusion of autologous, volume- and RBC-reduced, non-cryopreserved cord blood cells within the first few postnatal days was feasible and relatively safe in their cohort [49]. Five newborns (four with moderate and one with severe HIE) have undergone autologous UCBCs therapy without any significant adverse effects of treatment with survival up to one year [46]. While promising in its potential, larger well designed multi center trials are required to prove safety and efficacy prior to using this modality in standard practice.

### 5.4. Remote Ischemic Postconditioning (Endogenous)

The concept of remote ischemic conditioning (RIPC) involves delivery of sub-lethal small ischemic insults, remote from the area of injury, that activate endogenous repair pathways which potentially help in reducing the extent of original ischemic injury. This has previously been studied in adult cardiac injury [50], however is now being evaluated both in adult and neonatal brain ischemia and stroke [51]. RIPC, as it applies in neonatal HIE, would involve conditioned ischemic insults to the limb soon after the initial hypoxic ischemic insult. The ischemic skeletal muscle releases several endogenous compounds that activate not only the neuronal and humoral pathways, but also the systemic inflammatory response [52]. Delayed RIPC has significantly improved long term sensory motor deficits in a neonatal HI rat model [53]. In a piglet model of neonatal asphyxia, RIPC demonstrated a beneficial effect, mediated by modulation of nitrosative stress, despite glial activation [54]. Due to ease of application facilitating use even in low resource setting, this modality holds great promise both as an adjunct to therapeutic hypothermia, and also as a primary therapy in infants who may only have mild HIE and do not undergo cooling. Safety and efficacy of RIPC needs further evaluation in preclinical studies, especially in translational large animal models of neonatal HIE.

### 5.5. Endocannabinoids (Endogenous)

The endocannabinoid system has been recognized as an important neuroregulatory mechanism that could help in protection from brain injury. Activation of this system has been shown to decrease glutamate excitotoxicity and activation of microglia and cell death pathways. Use of a cannabinoid (CBD) receptor 1 and 2 (CBR1 and CBR2) agonist WIN 55212-2 in a rodent model of neonatal HIE demonstrated protective effects by prevention of glutamate release, TNF alpha accumulation, and iNOS induction, resulting in decreased cell death [55]. In another rodent model, WIN promoted neural stem cell proliferation in the sub ventricular zone and the short-term generation and migration of neuroblasts to the adjacent injured caudate [56]. Additionally, use of CBR agonists promotes oligodendrocyte generation, survival, and differentiation, thus contributing to repair from white matter injury [56]. In asphyxiated newborn piglets, intravenous administration of CBD improved brain oxygenation and induced a partial EEG recovery after HI [57]. As with other modalities describe above, while use of this therapy is promising, its safety and efficacy in preclinical studies continues to be evaluated.

### 5.6. Melatonin (Endogenous)

Melatonin is an endogenous neuroendocrine moiety secreted by the pineal gland and well known for its role in modulating the circadian rhythm. Besides this, melatonin has several other mechanisms that suggest an important role in recovery and repair from brain injury. Melatonin plays an important role in normal glial development [58] and has anti-apoptotic [59], anti-inflammatory, and anti-oxidant [60] effects. In a piglet model of neonatal HI injury, melatonin used along with hypothermia greatly decreased the HI-induced injury measured by magnetic resonance spectroscopy [61]. In a randomized controlled pilot trial evaluating melatonin with cooling in term infants with HIE, compared to controls, the HIE groups had increased serum melatonin and nitric oxide and plasma superoxide dismutase. The melatonin/hypothermia group also had fewer seizures, less evidence of white matter injury on MRI, and a lower rate of mortality without developmental or neurological abnormalities [62]. Melatonin has a good safety profile with low risk for toxicity [63,64] and thus holds tremendous promise in management of infants with HIE [65]. However, it has yet to be validated in larger randomized controlled trials.

### 5.7. Monosialoganglioside

Gangliosides are sphingolipids that serve an important function in maintaining cell membrane integrity. In a rat model of neonatal hypoxic ischemic injury, reduced ganglioside, phospholipid, and cholesterol contents in the hippocampus were noted [66]. Monosialoganglioside therapy has been shown to protect against apoptotic injury [67] and attenuate brain injury [68]. This led to consideration of monosialogangliosides as an adjuvant therapy in HIE. In a meta-analysis of all published clinical studies, Sheng et al. evaluated 10 trials consisting of 787 neonates. They concluded that adjuvant treatment with monosialoganglioside potentially offers additional benefits in terms of improving short-term clinical effects and reducing long-term neurodevelopmental disabilities [69]. However, they did acknowledge methodologic flaws in the included studies as well as limited information on optimal dosing, safety, as well as long term outcomes.

### 5.8. Xenon

Acute hypoxic ischemic insult leads to NMDA receptor activation through neuronal depolarization. Xenon inhibits NMDA signaling and thus may play a role in reducing the acute cell injury. While studies in the piglet model of birth asphyxia suggest a benefit to the combined modality of treatment with hypothermia and xenon [70,71], a proof of concept clinical trial (TOBY-Xe) [72] in 92 neonates concluded that xenon is unlikely to enhance the neuroprotective effects of cooling after birth asphyxia. Additionally, xenon is a noble gas that is expensive and requires a specialized delivery system [73]. Due to these factors, alternative therapies are currently being evaluated.

### 5.9. Argon

This is another significantly less expensive noble gas, that has demonstrated significant neuroprotection in animal models of HIE. In an extensive piglet model of ischemic injury, Broad et al. showed augmentation of hypothermic neuroprotection with argon use. They demonstrated reduced brain cell death, MRS, and aEEG improvements with combined argon hypothermia treatment [74]. Additionally, both xenon and argon have good blood brain barrier penetration [75].

### 5.10. Allopurinol

Oxidant injury by free radicals and superoxides formed through activation of the xanthine oxidase pathway contribute to the damage caused by a hypoxic ischemic insult. Allopurinol is a xanthine oxidase inhibitor that is being investigated as a potential agent for use in treatment of HIE. Preclinical studies in various rodent [76] and mammalian models [77] of HIE have shown neuroprotective effects with use alone and recently, as a complement to TH [78]. Only a few small trials in human neonates have been published so far and a Cochrane review in 2012 did not reveal any statistically significant difference in the risk of death or a composite of death or severe neurodevelopmental disability [79]. However a follow-up of two earlier performed randomized controlled trials at 4–8 years suggested a neuroprotective effect of neonatal allopurinol treatment in the subset of moderately asphyxiated infants [80]. Additional use of allopurinol antenatally in pregnancies at risk of hypoxic ischemic injury is being evaluated. A randomized blinded study of 53 pregnancies with evidence of fetal hypoxia demonstrated lower cord blood levels of S-100B, a short term marker of brain injury [81]; however, long term beneficial outcomes are yet to be proven conclusively.

### 5.11. Magnesium Sulfate

Magnesium sulfate is an NMDA receptor antagonist believed to reduce excitotoxic damage after a hypoxic ischemic insult. It is now being widely used antenatally for neuroprotection in preterm deliveries. Initial interest in use of this medication was generated due to low magnesium levels being noted in infants with HIE [82]. A prospective, longitudinal, placebo-controlled trial of MgSO_4_ use in infants with severe asphyxia, without hypothermia therapy, revealed encouraging short term outcomes compared to standard supportive treatment [83]. A systematic review of preclinical evidence for MgSO_4_ use in HIE noted inconsistent evidence of benefit as well as little consensus in dose and timing of administration [84]. Though generally considered a safe medication, there is some concern regarding risk of hypotension and bradycardia, especially with high doses of this medication which could limit its use [85]. A meta-analysis of five small randomized controlled trials evaluating MgSO_4_ in HIE concluded that there was improvement in short-term outcomes without significant increase in side effects [86]. However, there is a need for large well designed studies to determine if there are long-term benefits of magnesium and to confirm its safety, as a trend towards increased mortality was noted in the magnesium group [86]. Due to its low cost and ease of delivery, MgSO_4_ is thought to be a potentially viable therapy in low resource settings, in spite of its side effects [87].

### 5.12. Topiramate

Topiramate blocks the voltage-dependent sodium and calcium channels and also inhibits the excitatory glutamate pathway while enhancing the inhibitory effects of gamma-aminobutyric acid (GABA). All these effects would work favorably in the pathophysiology of HIE. In newborns, it has been extensively studied in the management of HIE in combination with hypothermia. The short-term and safety data support its use in combination with hypothermia in exploring the possible neuroprotective effects [88]. Although administration of topiramate in newborns with HIE is safe, it did not reduce the combined frequency of mortality and severe neurological disability [89]. The role of topiramate co-treatment in preventing subsequent epilepsy deserve further studies.

### 5.13. Azithromycin

Preclinical studies in models of ischemic stroke have revealed that azithromycin has a neuroprotective effect [90]. Recent abstracts have investigated the possibility of using azithromycin in neonatal HIE alone and as an adjunct to hypothermia [91]. This is a medication that has an established safety profile and is already being used in neonates, hence promising preclinical studies could quickly lead to clinical trials evaluating azithromycin as an adjunct to hypothermia therapy.

### 5.14. Combination Therapies

With the discovery of more therapeutic targets for management of HIE, there is potential for combination and adjunctive therapies with agents that may affect the pathophysiological process at different phases. Additionally, TH is being explored for mild HIE, as neurological deficits have been noted in these infants as well [92]. Melatonin, epo, darbe, xenon, and topiramate are all being studied as adjuncts to TH (Clinicaltrials.gov NCT02071160, NCT01913340, NCT01471015, NCT00934700, NCT01241019). Stem cells and Epo have actions that extend into the tertiary phase and thus may prove to be complementary to TH [93]. Creative ways of combining pharmacological and cell based therapies in secondary and tertiary phases of injury would be exciting and would especially benefit infants with HIE that evolved over time and missed the window for TH.

## 6. Management of Hypoxic Ischemic Injury beyond the Neonatal Period

Changes that occur in the brain after perinatal ischemic injury over time include loss of myelin, decreased neurogenesis, and reduced plasticity of the brain leading to tertiary brain damage [8]. These mechanisms contributing to tertiary brain damage may provide us with opportunities for long-term interventions in improving neurological function. Modulating persistent inflammation to promote neurogenesis is being experimented with IL-6 [94]. Imaging for gliosis, a function of chronic inflammation from activation of microglia [8], could be used as a diagnostic aid in the development of long-term treatments. Enhancing myelination by oligodendrocyte (ODC) stimulation could overcome ODC maturational arrest; with its ability to reduce perinatal inflammation and promoting white matter development [95,96].

Trichostatin-A, a histone deacetylase (HDAC) inhibitor has been shown to abolish behavioral and neuroendocrine deficits from early life stresses by promoting demethylation and possibly inducing epigenetic modifications [97]. HDAC inhibitors may restore loss of acetylation associated with inflammation and HI and improve and behavior outcomes in injury models in animals [98]. HDAC inhibitors are shown to modulate innate and humoral immune responses to enhance Th2 and TLR4 activation [99]. Modulation of innate of adaptive immune responses by lymphocyte activation is shown to reduce microglial activation and disease progression in animal models [100]. Administration of microRNAs (miRNA-124) to modulate microglial function and to improve neuronal function long after injury is also being explored as therapeutic targets [101].

Stem cell therapy offers enormous potential for stimulation of endogenous neural stem cells; to redirect new cell production from astroglia to oligodendrocytes and neurons following acute insult. Repeated treatments and delayed treatment in animal models has expanded the therapeutic window for treatment of tertiary brain damage by inducing repair and regeneration [102,103]. Activated microglia and macrophage phonotypes support neurogenesis and progenitor cell proliferation similar to stem cells, and hence are exciting candidates for repair and regeneration, as they migrate to site of injury and generate trophic factors [104]. Growth factors can prevent tertiary brain damage by attenuating inflammatory processes and promoting plasticity of the brain [105]. Ampakines, small and diffusible growth factors that easily diffuse across the blood–brain barrier and induce brain derived neurotrophic factor (BDNF) production in the brain when administered systemically are being explored [106].

Most of the therapies discussed above are studied in animal models with few current clinical studies, particularly in neonates with hypoxic ischemic encephalopathy. However, current research suggesting targets for therapy in infants with HIE in the tertiary phase, months to years after the acute insult offers hope to children with this devastating disease.

## 7. Limitations of Management in Resource Poor Settings

Therapeutic hypothermia, while standard of care in developed countries, remains an expensive and sometimes unaffordable modality of treatment in resource limited settings. Practical difficulties are often encountered in transferring patients, often in rural settings to tertiary care centers within the 6-h window for therapeutic hypothermia. An additional concern initially identified in a randomized controlled trial of TH by Robertson et al. was the high mortality rate that was noted in the enrolled infants [107]. A systematic review and meta-analysis of trials evaluating TH for neonatal encephalopathy in low-and middle-income countries (LMIC) also showed that TH was not associated with a statistically significant reduction in neonatal mortality [108]. Major contributing factors, besides lack of tertiary care facilities and adequate neonatal transport, were thought to be the high rate of neonatal sepsis, inflammation and growth retardation [109] suggesting a chronic perinatal insult in these infants in LMIC. Rodent models primed with infection prior to a hypoxic ischemic insult have demonstrated reduced protection with hypothermia [110]. Thus, with current limitations in LMIC, alternative low cost cooling methods as well as adjuncts and affordable treatment options such as magnesium are being evaluated [87].

## 8. Conclusions

Birth asphyxia contributes to a significantly higher burden of neonatal mortality and morbidity globally, more so in developing countries. Neonatal HIE, apart from increased mortality leads to devastating neurological consequences such as cerebral palsy, epilepsy, and mental retardation. With the advent and widespread clinical use of TH over the last decade, the prognosis of moderate HIE has significantly improved. However, TH is expensive, needs a multidisciplinary team and is further limited by narrow therapeutic window for initiation. With issues of access to health care and the burden of birth asphyxia shifting to developing and least developed nations, there is a need for alternative and supplementary neuroprotective agents. Low cost and easy availability along with ease of use would assist in ensuring that these therapies have global applicability. Several of the promising modalities we have described in this review are still in the preclinical stage. It is uncertain how many of these therapies, alone or in combination, will achieve widespread use in the management of neonatal HIE. Large well designed randomized control trials in developed countries as well as in low resource settings, are required to prove the safety and efficacy of these therapies, prior to widespread clinical use. Neonatal HIE remains a condition of global importance with significant disparities in available treatment options among developed and developing countries. Hence, a concerted effort to address this problem on a global scale is essential.

## Figures and Tables

**Table 1:** Promising therapies in management of HIE by mechanism.

Mechanism	Clinical Trials	Pre-Clinical Studies
Endogenous	Erythropoietin, darbepoietin	Remote ischemic postconditioning
	Stem cells	Endocannabinoids
	Melatonin	
Exogenous	Monosialogangliosides	Argon
	Xenon	Azithromycin
	Allopurinol	
	Topiramate	
	Magnesium sulfate	
